# A Novel Therapeutic Peptide as a Partial Agonist of RANKL in Ischemic Stroke

**DOI:** 10.1038/srep38062

**Published:** 2016-11-29

**Authors:** Hitomi Kurinami, Munehisa Shimamura, Hironori Nakagami, Hideo Shimizu, Hiroshi Koriyama, Tomohiro Kawano, Kouji Wakayama, Hideki Mochizuki, Hiromi Rakugi, Ryuichi Morishita

**Affiliations:** 1 Department of Health Development and Medicine, Osaka University Graduate School of Medicine, Japan; 2 Postgraduate Medical Training Centre, Osaka University Hospital, Japan; 3 Department of Neurology, Osaka University Graduate School of Medicine, Japan; 4 Department of Nutritional Science, Kansai University of Welfare Sciences, Japan; 5 Department of Advanced Clinical Science and Therapeutics, Graduate School of Medicine, the University of Tokyo, Japan; 6 Department of Geriatric and General Medicine, Osaka University Graduate School of Medicine, Japan; 7 Department of Clinical Gene Therapy, Osaka University Graduate School of Medicine, Japan

## Abstract

The enhanced receptor activator of nuclear factor-κB (NFκB) ligand (RANKL) and its receptor (RANK) signal have been reported to attenuate ischemic brain injury through inhibition of Toll-like receptor (TLR) 4-mediated inflammation. However, augmentation of the RANKL/RANK signal also accelerates osteoporosis, which is a potential problem in clinical use of RANKL. Therefore, we developed novel peptides, microglial healing peptides (MHPs), which were based on the DE and/or EF loop of RANKL. Among them, MHP1 was the most effective inhibitor of TLR4-induced inflammations in microglia/macrophages. The effects depended on RANK, as confirmed by knockdown experiments. In contrast to RANKL, MHP1 did not stimulate osteoclast differentiation. Unexpectedly, MHP1 inhibited RANKL-induced osteoclast differentiation. These findings suggested that MHP1 was a partial agonist of RANKL, and administration of MHP1 attenuated ischemic injury by decreasing inflammation. MHP1 could be a novel therapeutic agent for treating ischemic stroke.

Regulation of post-ischemic inflammation is an important strategy for treating ischemic stroke^1^. However, most recent clinical trials targeting post-ischemic inflammation, including SUN-N8075^2^, minocycline^3^ and uric acid^4^, have failed to show efficacy. Although edaravone is the only free radical scavenger accepted in Japan, China and India, its effectiveness has not been shown in large high-quality trials^5^. Consequently, novel signalling processes that control post-ischemic inflammation have been explored to develop new therapeutic approaches. Among these approaches, we have recently found that the receptor activator of nuclear factor-kB (NFκB) ligand (RANKL)/receptor activator of NFκB (RANK) is a novel signal involved in the regulation of microglial inflammation through Toll-like receptor (TLR) 4^6^, which is a main damage-associated molecular pattern (DAMP) receptor in the ischemic brain^1^. Both RANKL and RANK are expressed in activated microglia and macrophages (M/M) of ischemic brain tissue, and enhancement of the RANKL/RANK signal using recombinant RANKL (rRANKL) has been shown to reduce ischemic injury in mice^6^; this indicated that rRANKL could potentially be used as a therapeutic agent for treating ischemic stroke.

However, a potential problem is that RANKL and RANK are expressed in osteoclast precursors and have been found to be key molecules, inducing osteoclast differentiation^7^. A recent study showed that systemically administered rRANKL stimulated osteoclast differentiation and caused bone loss with a minimum of three rRANKL i.p. injections at 24-h intervals^8^, which indicated that systemic administration of rRANKL might exacerbate osteoporosis. To address this unfavourable action of RANKL, we investigated the region of RANKL that was responsible only for the inhibitory effects on TLR-mediated inflammation without affecting osteoclast differentiation.

Structurally, the binding sites of RANKL at its receptor, RANK, have been reported to be at the AA″, CD, DE and EF loops^9^. Experiments using RANKL mutants have shown that the AA′′^9^ or AA′′/CD loops^10^ are the core regions that activate RANK signal-induced osteoclast differentiation^9^. RANKL mutants (aa239–318) that include the DE and EF loops show much less osteoclast differentiation, whereas approximately half of the downstream signal of RANK, NFκB, is preserved compared to that of the mutant with the AA′′/CD/DE/EF loops^9^. From these previous reports, we hypothesized that the DE and/or EF loop-based peptides suppress TLR-mediated inflammation without the induction of osteoclast differentiation; however, the association of activated NFκB with decreased TLR-mediated inflammation in RANKL/RANK signal is controversial.

To test this hypothesis, we designed several types of DE and/or EF loop-based partial peptides, namely microglial healing peptides (MHP), and examined the anti-inflammatory effects of these peptides in cultured M/M and the effects on osteoclast differentiation in osteoclast precursor cells. In addition, we examined the effects of MHP in the ischemic stroke model in mice to assess the potential of the peptide for treating ischemic stroke.

## Results

**Figure 1:**
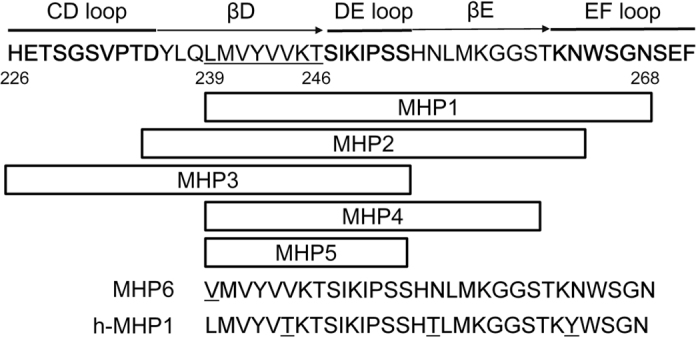
Structure of microglia-healing peptides (MHP). The structure of the region of the CD loop, DE loop and EF loop in mouse RANKL^9^ is shown. MHP1 and −2 included the DE loop and a part of the EF loop, MHP3 contained the CD and EF loops and MHP4 and 5 included the DE loop. MHP6 is modified MHP1 in which the N-terminal leucine was changed to valine (the underlined amino acid). Human MHP1 (h-MHP1) was designed from human RANKL. The underlined amino acids are different from those in mice.

**Figure 2:**
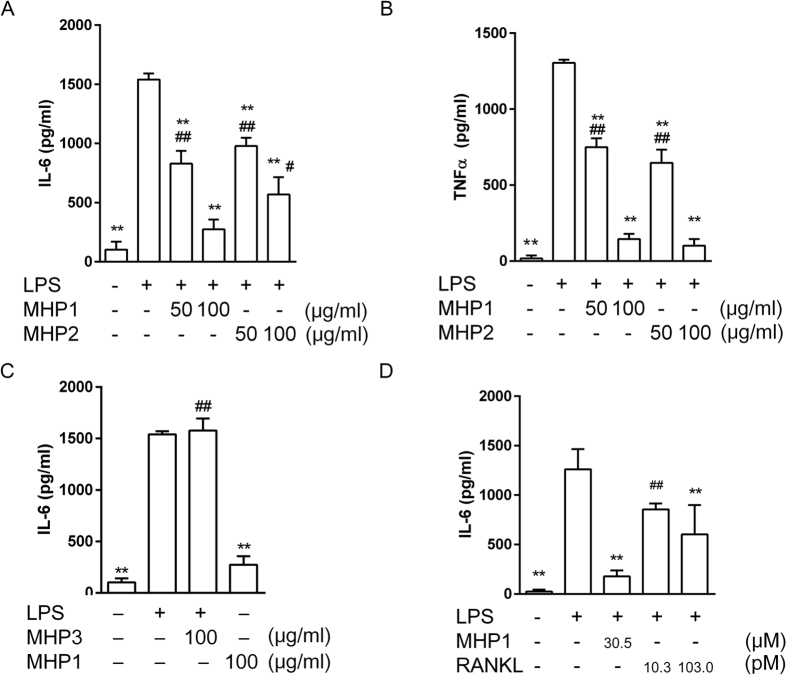
Effects of MHP1 and MHP2 in LPS-stimulated MG6 cells. Cytokine expressions in MG6 cells treated with LPS and MHP1 (**A–D**), MHP2 (**A,B**), MHP3 (**C**) or recombinant RANKL (**D**). MHP1 and 2 inhibited expression of IL-6 and TNF-α, but MHP1 was more effective than MHP2 for IL-6 (**A**). In contrast, MHP3 showed no effects (**C**). When the effects of MHP1 (100 μg/ml, 30.5 μM) were compared with those of recombinant RANKL (rRANKL), the effects were comparable to those in rRANKL (**D**). The concentration of rRANKL was similar to those in previous reports that used bone marrow-derived macrophages (10 ng/ml, 10.3 pM)^12^ and a primary mixed neuron-glia culture (100 ng/ml, 103.0 pM)^6^. **P < 0.01 vs. cells treated with LPS; ^##^*P* < 0.01 or ^#^*P* < 0.05 vs. MHP1 (100 μg/ml)-treated cells. N = 3 in each group.

**Figure 3:**
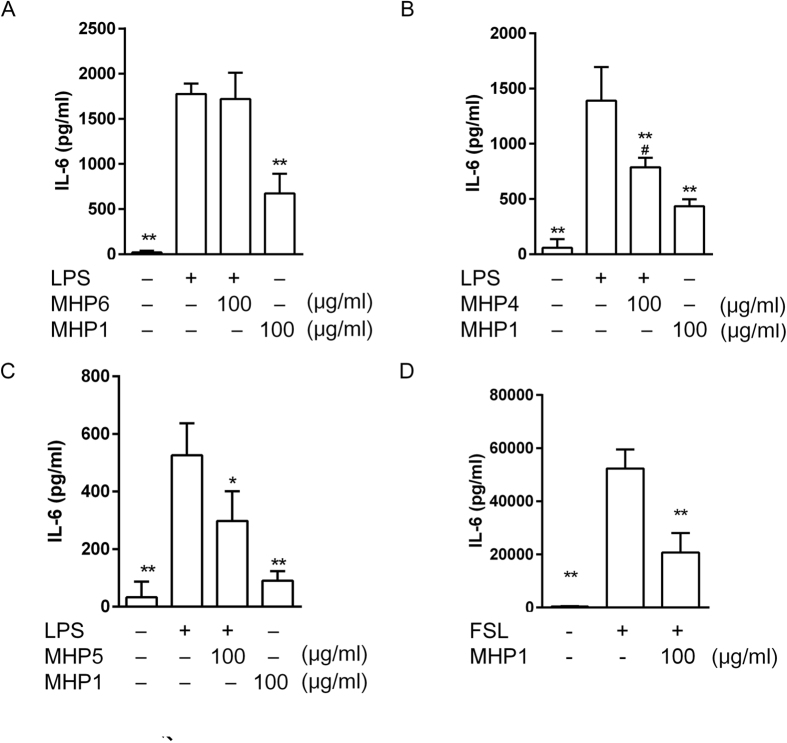
The effect of MHP1, 4–6 on IL-6 expression in LPS or FSL-1-stimulated MG6 cells. The effects of MHP1 (**A–D**), MHP6 (**A**), MHP4 (**B**) and MHP5 (**C**) on IL-6 expression in LPS-stimulated MG6 cells (**A–C**) or FSL-1-stimulated MG6 cells (**D**). MHP6 did not show any effects (**A**). MHP4 and 5 were less effective than MHP1, but significantly reduced IL-6 expression in LPS-stimulated MG6 cells (**B,C**). FSL-1-induced IL-6 was reduced in the MHP1-treated cells (**D**). *P < 0.05, **P < 0.01 vs. the LPS or FSL-1 -treated group, ^#^*P* < 0.05 vs. the MHP1-treated group. N = 5 (**A,B,D**) or 3 (**C**) in each group.

Initially, we designed MHP1 and MHP2, which included the DE loop and part of the EF loop (Fig. 1); we examined whether these peptides would produce inhibitory effects on TLR4-mediated inflammation using the microglial cell line, MG6. MHP1 and MHP2 showed significant inhibitory effects on production of LPS-induced cytokines, including interleukin-6 (IL-6) and tumour necrosis factor α (TNF-α, Fig. 2A,B). MHP1 was a more effective inhibitor of IL-6 production than MHP2 (Fig. 2A). In contrast, MHP3, which was designed to include both the CD and DE loops, showed no inhibitory effects (Fig. 2C). Based on these results, we further focused on the most effective peptide, MHP1, in subsequent experiments. When the anti-inflammatory effects of MHP1 were compared with those of rRANKL, whose dose were equivalent to those mentioned in previous reports^6^,^11^, the effects were comparable to those in rRANKL (Fig. 2D). To confirm that cell death did not cause the inhibitory effects of MHP1, we examined the number of cells present 24 h after the treatment. There was no decrease in the numbers of cells in the cultures treated with MHP1 and LPS (82.2 ± 11.9 cells/field in the control; 68.7 ± 5.9 cells/field in LPS-treated cells; 85.7 ± 7.8 cells/field in MHP1 and LPS-treated cells, N = 6 in each group), which indicated that the anti-inflammatory effects were not due to cell death. Next, we tried shortening of MHP1. When the N-terminal leucine was changed to valine (MHP6), the anti-inflammatory effect was completely lost (Fig. 3A). MHP4 and MHP5, which comprised 23 and 15 amino acids, respectively, achieved by truncation of the C-terminus in MHP1 (Fig. 1), were less effective than MHP1 (Fig. 3B,C). These data indicated that the N-terminus was critical for the activity of MHP1, but the C-terminus could be truncated by at least 15 amino acids and still retain some activity.

Because macrophages as well as microglia are important targets for treating ischemic stroke, we also examined the efficacy of MHP1 in RAW 264.7 cells. As expected, MHP1 suppressed the LPS-induced inflammatory cytokines, IL-6 and TNF-α in a dose-dependent manner (Supplementary Fig. S1). Furthermore, we examined whether MHP1 was also effective in the human monocyte cell line, THP1. For this experiment, h-MHP1, which differs by three amino acids from MHP1 (Fig. 1), was used. MHP1 significantly decreased production of IL-6 in the THP1 cells to the same extent as did h-MHP1 (Supplementary Fig. S1). This result suggested that h-MHP1 was effective in human cells; however, MHP1, which was designed from mouse RANKL, could be potentially effective in humans.

**Figure 4:**
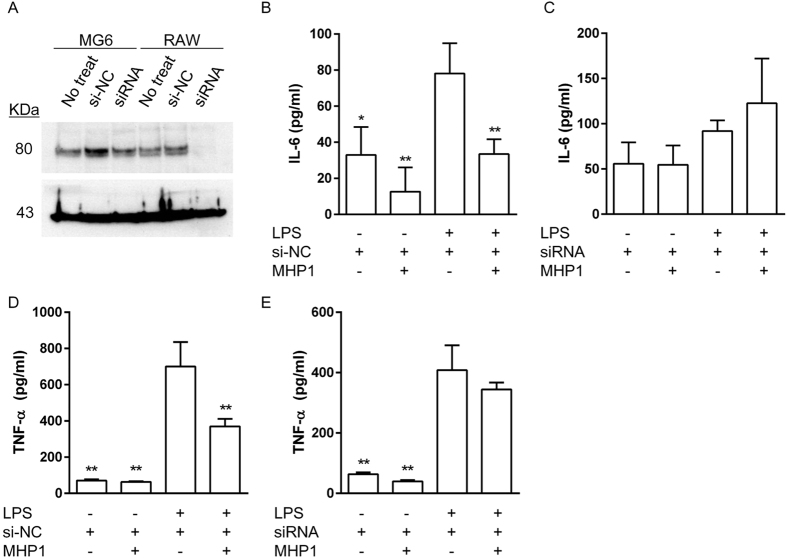
Influences of RANK knockdown on the expression of inflammatory cytokines in RAW 264.7 cells. MG6 or RAW 264.7 cells were treated with RANK-specific siRNA or the negative control siRNA (**A**). In the negative control siRNA-treated RAW 264.7 cells, MHP1 inhibited expression of IL-6 and TNF-α (**B**,**D**). In contrast, their expression levels were not reduced by MHP1 in the RANK-specific siRNA-treated cells (**C**,**E**). si-NC: Negative control for RANK siRNA; siRNA: RANK siRNA. *P < 0.05, **P < 0.01 vs. LPS without MHP1-treated group. N = 3 in each group.

Next, we explored whether this inhibitory effect of MHP1 was dependent on RANK using cells in which RANK had been knocked down with siRNA. Expression of RANK was successfully reduced in RAW 264.7 cells by siRNA (Fig. 4A). Quantitative analysis showed that the ratio of band density normalized to β-actin was 0.60 ± 0.18 or 0.13 ± 0.09 in negative control siRNA or RANK-siRNA, respectively, indicating that knockdown efficiency was 70.8 ± 8.3%. Although MHP1 significantly inhibited LPS-induced IL-6 and TNF-α expression in cells treated with the negative control siRNA (Fig. 4B,D), the expression levels of IL-6 and TNF-α were not changed in the RANK-siRNA-treated cells (Fig. 4C,E). These results indicated that MHP1 inhibited the LPS-induced inflammatory cytokines through RANK-induced signals.

Because TLR2 is another important receptor for DAMPs in the ischemic brain^1^ and other studies have shown that RANKL inhibited the TLR2 signal^12^,^13^, we further investigated whether MHP1 could inhibit TLR2-stimulated signalling in MG6 cells. When the MG6 cells were treated with MHP1, TLR2 (FSL-1)-mediated inflammation was also inhibited successfully (Fig. 3D). To exclude the possibility that the anti-inflammatory effects of MHP1 were nonspecific, we checked the effects of MHP1 on S100β-stimulated MG6 cells. MHP1 did not inhibit the expression of IL-6 in cultured medium (21.8 ± 7.7 pg/ml in control; 99.1 ± 4.7 pg/ml in S100β-treated cells; 135.1 ± 25.3 pg/ml in MHP1 and S100β-treated cells, N = 3 in each group), which indicated that the effects of MHP-1 were not nonspecific.

**Figure 5:**
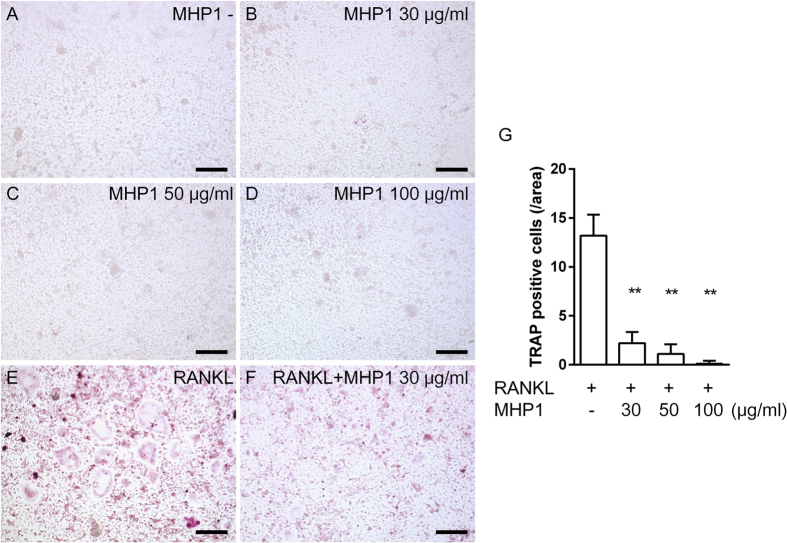
Inhibitory effects of MHP1 on RANKL-induced osteoclast differentiation in primary osteoclasts. Images of TRAP staining (**A–F**, Bar = 200 μm). The osteoclast precursor cells were treated with M-CSF (**A**), M-CSF + MHP1 (**B–D**), M-CSF + rRANKL (**E**) or M-CSF + rRANKL + MHP1 (**F**). Osteoclast precursor cells were differentiated into osteoclast cells after treatment with rRANKL (**E**), whereas MHP1 did not induce osteoclast differentiation (**B–D**). Interestingly, MHP1 inhibited RANKL-induced osteoclast differentiation (**F**) in a dose-dependent manner (**G**). **P < 0.01 vs. cells treated with rRANKL alone. N = 10 in each group.

To check whether MHP1 could affect osteoclast differentiation, we next examined the effects of MHP1 on primary osteoclast precursor cells (Fig. 5). Although the osteoclast precursor cells treated with macrophage colony-stimulating factor (M-CSF) and rRANKL differentiated into osteoclasts (Fig. 5E), treatment with M-CSF and MHP1 at the concentration used for the inhibitory effects on TLR4-related inflammation did not induce osteoclast differentiation (Fig. 5B–D). Unexpectedly, MHP1 also blocked RANKL-induced osteoclast differentiation in a dose-dependent manner (Fig. 5F,G).

**Figure 6:**
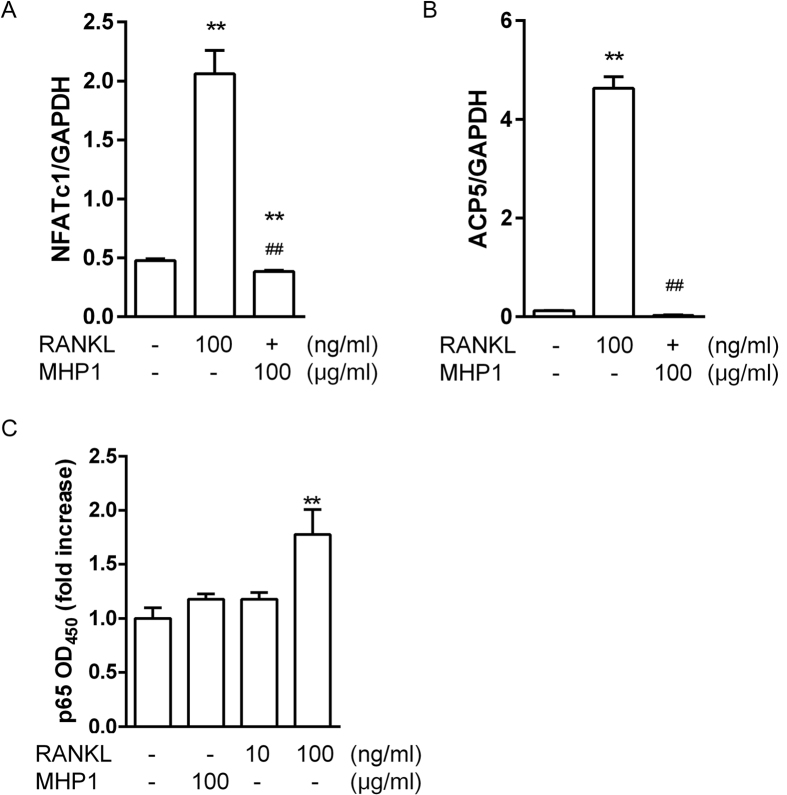
Comparison of effects of MHP1 and rRANKL on *NFATc1, ACP5* mRNA and nuclear p65 expression. *NFATc1* or *ACP5* mRNA in RAW264.7 cells cultivated with rRANKL or MHP1 for 3 days (**A,B**) and expression of nuclear p65 at 10 min after cultivation (**C**). **P < 0.01 vs. non-stimulated cells, ^##^*P* < 0.01 vs. cells treated with rRANKL. N = 3 in each group.

Thus, the inhibitory effects of TLR2 and TLR4 signals in MHP1 were similar to those in rRANKL, but the actions on osteoclast differentiation were different between MHP1 and rRANKL. To clarify the mechanism, we examined the expressions of downstream signals of RANK, including *NFATc1* mRNA (a master regulator of osteoclast differentiation), *ACP5* mRNA (an osteoclast marker) and nuclear translocation of NFκB (p65). When RAW264.7 cells were stimulated with rRANKL or MHP1 for three days to induce osteoclast differentiation, the expressions of *NFATc1* mRNA and *ACP5* mRNA increased in rRANKL but not in MHP1 (Fig. 6A,B). Translocation of p65 was not significantly increased in MHP1-treated RAW264.7 cells, although the expression level was increased in cells treated with rRANKL at 100 ng/ml (Fig. 6C). These results indicated that MHP1 did not activate the NFκB signal and the signal for osteoclast differentiation.

**Figure 7:**
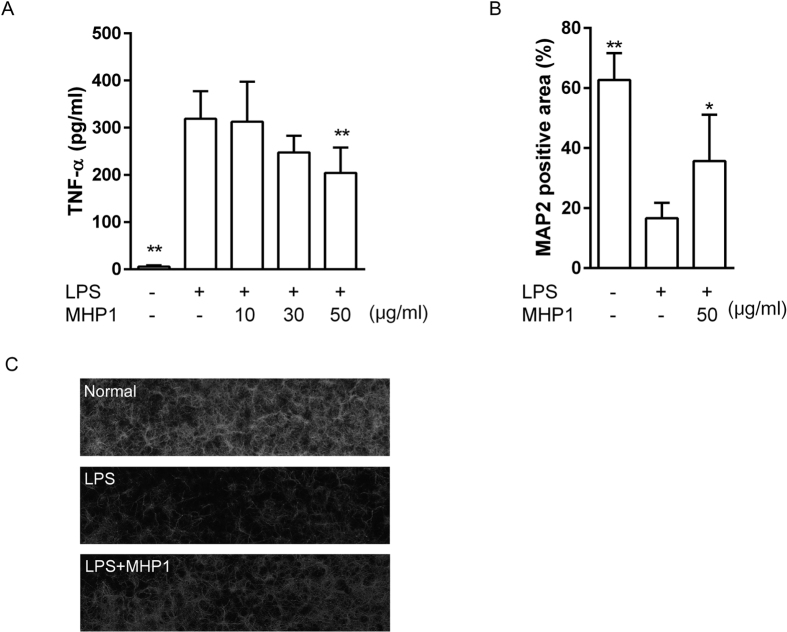
Anti-inflammatory effects of MHP1 on LPS-triggered neuronal death in primary mixed neuronal-glial cultures. (**A**) The cells were pre-treated with MHP1 for 24 hours, and LPS was added to the medium. During LPS stimulation, MHP1 treatment of the cells was continued. Expression of TNF-α was inhibited in cells treated with MHP1. (**B**) Quantitative analysis of the surviving neurons at 5 days after LPS stimulation. (**C**) Typical images of the surviving MAP2-positive neurons at 5 days after LPS stimulation. The addition of LPS caused neuronal death (in the middle panel), whereas pre-treatment with MHP1 prevented neuronal death (in the lower panel). *P < 0.05, **P < 0.01 vs. LPS without MHP1-treated cells. N = 5 in each group.

Next, we examined the effects of MHP1 in primary mixed cultures of neurons and glia. Because we have previously shown that LPS-induced expression of TNF-α was decreased by 24-h pre-treatment treatment with rRANKL^6^, we evaluated the effects under this condition. As expected, MHP1 significantly reduced LPS-induced expression of TNF-α (Fig. 7A) and prevented delayed neuronal death induced by LPS (Fig. 7B,C).

**Figure 8:**
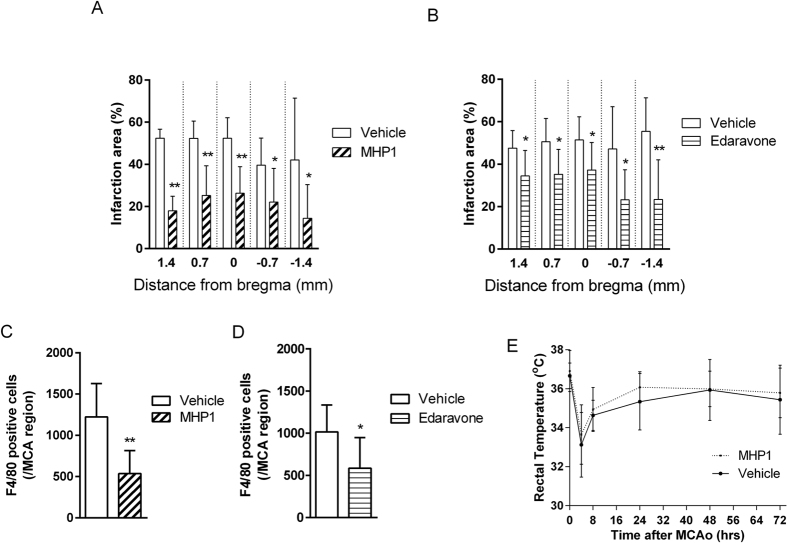
Effects of intracerebroventricular injection of MHP1 on the ischemic brain. Infarction areas at 72 hours after the ischemic-reperfusion insults. (**A,B**) MHP1 (i.c.v.) or edaravone (i.p.) was injected 4 hours after middle cerebral artery occlusion. Edaravone was continued to be injected twice per day. The infarction areas in the MHP1 or edaravone-treated mice were less than those in vehicle control mice. (**C,D**) Fewer F4/80^+^ activated macrophages in the region in the middle cerebral artery were observed in the MHP1 or edaravone-treated mice. (**E**) There were no differences in the rectal temperatures between the MHP-1 and vehicle control mice. *P < 0.05 and **P < 0.01 vs. vehicle group. N = 8 in each group.

Finally, the effects of MHP1 in an ischemic brain model were examined to explore the therapeutic potential. Because previous studies showed that i.c.v. injection of the synthetic peptide, which was effective in 1 μM^14^ or 12 μM^15^ in cultured cells, was also effective in brain in 2 μg/rat^14^ or 5 μg/rat^15^, we speculated that MHP1, which showed its effectiveness from 50 μg/ml (15.3 μM) to 100 μg/ml (30.5 μM), might be effective in around 1 μg/mice in ischemic brain model. In a preliminary study, we intracerebroventricularly injected MHP1 at 1.25 μg, 0.625 μg, 0.313 μg, or vehicle 4 hours after an ischemia and found that the infarct area at 0 mm from the bregma was 64.7%, 22.6%, 46.4%, 63.5%, respectively. Based on this result, we decided to inject 0.625 μg/mice. A single i.c.v. injection of MHP1 at 4 hours after an ischemic insult significantly decreased the infarct areas and the number of F4/80-positive activated M/Ms (Fig. 8A,C). There were no differences in rectal temperature between the groups (Fig. 8E). We also checked whether MHP1 had influences on the body temperature because a previous study showed i.c.v. injection of recombinant RANKL caused fever from 4 hours after injection in normal mice^16^. A single i.c.v. injection of MHP1 in normal mice did not affect the body temperature (Supplementary Fig. S2). These data indicated that the therapeutic effect of MHP1 was not due to its actions on body temperature. To check whether the decrease of F4/80-positive activated M/Ms was specific in MHP1-treated mice, we examined the effects of edaravone in the same model. Because edaravone also reduced the infarct area and F4/80 positive M/Ms (Fig. 8B,D), the reduced number of F4/80 positive M/Ms in MHP1-treated mice was not due to specific effects of MHP1.

In preparation for clinical trials, the stability of MHP1 in long-term storage at 4 °C was further examined (Supplementary Fig. S3). MHP1 dissolved in ddH_2_O (concentration, 1 mg/ml) that had been stored at 4 °C for 6 months was added to the culture medium. Expression of inflammatory cytokines was still significantly inhibited by MHP1 treatment (Supplementary Fig. S3). These data confirmed the long-term stability of MHP1.

## Discussion

In this study, we demonstrated that the novel peptide, MHP1, which was designed from RANKL, significantly prevented exacerbation of ischemic brain injury *in vivo*. This effect was associated with inhibition of TLR2- and TLR4-induced inflammation in cultured M/M through its effects on RANK. Despite these anti-inflammatory effects of MHP1, the peptide did not induce osteoclast differentiation and osteoclast-related signals different from those of RANKL. Thus, MHP1 can be considered as a partial agonist of RANKL.

Because the anti-inflammatory effects of MHP1 depended on RANK, we speculated that the mechanisms underlying the anti-inflammatory effects of MHP1 were similar to those of RANKL/RANK signal; however, the mechanisms of anti-TLR signal effects of RANKL/RANK signal are controversial. For example, one study using bone marrow macrophages (BMMs) has reported that RANKL inhibited *Porphyromonas gingivalis*-induced cytokine production by downregulation of TLR/NF-κB and upregulation of NFATc1^13^. The authors mentioned that robust induction of NFATc1 by RANKL negatively regulated NF-κB activity, which resulted in reduced TLR-mediated cytokine production^13^. In contrast, another study demonstrated that the inhibitory effects of RANKL for TLR4 signalling occurred independently of the differentiation of BMMs into osteoclasts^12^. In our experiments using RAW264.7 cells, RANKL activated NF-κB and NFATc1, whereas MHP1 did not activate NF-κB and NFATc1, but was able to inhibit TLR4-mediated cytokine production through RANK. These results suggest that the activation of NF-κB is not necessary for the anti-TLR4-mediated cytokine production in RANKL/RANK signal, although further studies are necessary to confirm this speculation.

It is possible that the effects of MHP1 depend on direct binding to TLR ligands. For example, the LL-37 peptide, a 37-residue antimicrobial peptide produced by human epithelial cells, suppresses TLR4 signalling by binding to multiple TLR agonists, including lipopolysaccharides^17^. However, this mechanism is unlikely for MHP1 because the inflammatory cytokines did not decrease in the RANK-deficient cells treated with LPS and MHP1. In addition, the lack of anti-inflammatory responses in S100β-stimulated MG6 cells supports the idea that nonspecific binding of MHP1 to ligands may not be unlikely.

MHP1 is interesting not simply because of its lack of osteoclast activation but rather because of its inhibitory effects on RANKL-induced osteoclastic differentiation. The reason for this effect is probably because MHP1, which lacks the AA′/CD loop required for osteoclast differentiation, binds to RANK and may compete with RANKL. Considering that the anti-inflammatory and anti-osteogenic properties of MHP1 were observed at the same concentration, MHP1 is similar to the partial agonist, aripiprazole, which activates the D2 receptor but blocks the action of dopamine at the same concentration^18^. This property is important clinically because osteoporosis is one of the complications associated with poor prognosis in ischemic stroke. Ischemic stroke patients have an approximate 2.0-fold increase in the risk of hip/femur fracture^19^, and recent studies have reported that the biochemical markers of bone resorption increase within 7 days after the onset of ischemic stroke^20^. In experiments in rats, activation of osteoclasts has also been reported to start 7 days after ischemic stroke, and RANKL secreted from the post-ischemic activated T cells was considered to be one of the causes^21^. Thus, regulation of osteoporosis as well as inflammatory cytokines in the brain are important in the treatment of ischemic stroke, but previously reported peptides, such as thymosin β4 (Tβ4)^22^, synthetic fibronectin peptides^23^,^24^, death-associated protein kinase 1^25^ or laminin peptides^26^, did not affect the M/M or osteoclast differentiation. Consequently, the anti-inflammatory and anti-osteoclast effects of MHP1 indicate that it could potentially be a unique therapeutic agent in ischemic stroke.

The cerebroprotective effects of i.c.v. administration of MHP1 have been demonstrated in a stroke model in mice, but systemic administration should be further examined for clinical use. However, in general, the synthetic peptides were easily degraded by peptidases in blood, and some modifications are needed to stabilize them, although the degradation pattern of MHP1 in blood should be analysed by high-performance liquid chromatography and liquid chromatography/mass spectrometry. Considering that the anti-inflammatory activity in MHP4 (C-terminal truncation of MHP1) remained and was completely inactivated in MHP6 (N-terminal displacement of MHP1), modification of peptides for preservation of N-terminal amino acids, such as substitution with D-amino acids, is critical for maintaining the activity of MHP1 in systemic administration.

Overall, this study showed that the novel synthesized peptide, MHP1, which included the DE loop and a part of the EF loop, significantly inhibited TLR2- and TLR4-induced inflammatory responses and RANKL-induced osteoclast differentiation. Because regulation of post-ischemic inflammation in the brain and osteoclast activation in the bone are important in the treatment of ischemic stroke, targeting RANK signalling with this novel dually-effective peptide could be a promising approach, although further studies are needed to elucidate the molecular mechanisms of the anti-TLR2 and anti-TLR4 signalling as well as to discover how to stabilize the synthetic peptide for systemic administration.

## Methods

### Peptide design and synthesis

Synthetic MHP1 (NH2-LMVYVVKTSIKIPSSHNLMKGGSTKNWSGN-COOH), MHP2 (NH2-DYLQLMVYVVKTSIKIPSSHNLMKGGSTKN-COOH), MHP3 (NH2-HETSGSVTDYLQLMVYVVKTSIKIPSS-COOH), MHP4 (NH2- LMVYVVKTSIKIPSSHNLMKGGS-COOH), MHP5 (NH2-LMVYVVKTSIKIPSS-COOH), MHP6 (NH2-VMVYVVKTSIKIPSSHNLMKGGSTKNWSGN-COOH) and h-MHP1 (NH2-LMVYVTKTSIKIPSSHTLMKGGSTKYWSGN-COOH) were purchased from ILS, Inc. (Ibaragi, Japan).

### Cell culture and enzyme-linked immunosorbent assay (ELISA)

MG6 cells were obtained from RIKEN BRC, Japan, RAW 264.7 cells were obtained from ECACC, UK and THP1 cells were purchased from American Type Culture Collection (Rockville, MD, USA).

The MG6 cells were maintained in DMEM (Nakarai, Kyoto, Japan) supplemented with 10% FBS (Thermo Fisher Scientific, Waltham, MA, USA), 10 μg/ml insulin (Sigma-Aldrich, St. Louis, MO, USA) and 100 μM 2-mercaptoethanol (Sigma-Aldrich), and the RAW264.7 and THP1 cells were maintained in DMEM supplemented with 10% FBS. These cells (4 × 10^4^ cells) were plated in 24-well plastic culture dishes. After overnight culture, the medium was replaced with DMEM supplemented with 4% FBS. LPS (*Escherichia coli* 0111:B4; Sigma-Aldrich, St. Louis, MO, USA) and MHP were added to the medium, which was then harvested at 24 hours after stimulation. The concentrations of TNF-α and IL-6 were measured using commercially available ELISA kits: TNF-α, Quantikine Mouse TNF-α ELISA Kit (R&D systems); IL-6, Quantikine Mouse IL-6 ELISA Kit (R&D systems).

The primary neuron-glial cultures were prepared from C57BL/6J mice on post-natal days 1–2, as previously described^6^. Briefly, the entire cerebral cortex was dissected and minced in Neurobasal-A (Invitrogen, San Diego, CA, USA) with B-27 supplement (Neurobasal-A/B-27; Invitrogen) after removal of the meninx. The cells were treated with papain (25 U/ml) and DNaseI (25 U/ml) for 30 min at 30 °C. The cells were dissociated mechanically in Neurobasal-A/B-27 with 0.25 mM Glutamax (Invitrogen) and 10% horse serum using a siliconized Pasteur pipette. The cells were plated on polyethyleneimine-coated 24-well plates at 7 × 10^5^/well. The cultures were maintained at 37 °C in a humidified atmosphere containing 5% CO_2_. Half of the medium was replaced with Neurobasal-A/B-27 containing 5% horse serum twice per week. After 9 days of culture, the cells were treated with MHP1 (1–50 μg/ml) for 24 hours, and LPS (10 μg/ml) was added to the medium. In the experiment to examine the expression of TNF-α or IL-6, the medium was collected at 24 hours after LPS stimulation. To assess the neuronal death, the cells were immunostained with MAP-2 (Sigma-Aldrich) to determine the survival of the neurons 5 days after the addition of LPS. The images were then digitized using a microscope (FSX-100; Olympus, Tokyo, Japan). The acquired images were converted to grayscale with GIMP 2.8.6 (available at http://www.gimp.org website), and the stained area was calculated using ImageJ (National Institutes of Health).

### siRNA-mediated gene knockdown in cultured cells

The following were purchased from Gene Design (Osaka, Japan): siRNAs to target murine RANK (sense 5′-GCGCAGACUUCACUCCAUAUU-3′, antisense 5′-UAUGGAGUGAAGUCUGCGCUU-3′) and a non-targeting control siRNA (sense 5′-UAGCGACUAAACACAUCAAUU-3′, antisense 5′-UUAUCGCUGAUUUGUGUAGUU-3′). HiPerFect Transfection Reagent (Qiagen, Germantown, MD, USA) was used as the cationic lipid cell transfection reagent. The RAW 264.7 cells were plated at a density of 4 × 10^4^ cells per well in 24-well plastic culture dishes in DMEM, and siRNA transfection was performed immediately. The transfected cells were incubated at 37 °C with 5% CO_2_ for 24 hours. The cells were lysed in RIPA buffer, and the concentration of the obtained protein extract was measured by DC^TM^ Protein assay (Bio-Rad, Hercules, CA, USA). The protein extracts were size-fractionated using sodium dodecyl sulphate-polyacrylamide gel electrophoresis and transferred to a PVDF membrane. Blotting was performed using anti-RANK antibody (1:100; R&D Systems). Images of blots were acquired using Kodak digital imager. Densitometry was performed with ImageJ software (National Institutes of Health) and the RANK band intensity was normalized to β-actin.

### Real-time reverse transcription polymerase chain reaction (RT-PCR)

RAW 264.7 cells (1 × 10^4^) cells were plated onto 24-well plates. rRANKL or MHP1 was added to the medium 24 hours after plating, and the cells were collected. The mRNAs were isolated using QIAGEN RNeasy Mini Kit (Qiagen) according to the manufacturer’s recommendations. The cDNA reaction was performed using a High-Capacity cDNA Archive kit (Applied Biosystems) according to the manufacturer’s instructions. The oligonucleotide primers used exclusively in the *in vitro* experiments were purchased according to the following identification: *NFATc1*, Mm00479445_m1; *ACP5*, Mm00475698_m1 and *GAPDH*, Mm99999915 (Applied Biosystems). The 5′ nuclease assay PCRs were performed in a MicroAmp Optical 384-well reaction plate using an ABI PRISM 7900 Sequence Detection System. The levels of the target genes were quantified by comparing the fluorescence generated by each sample with that of the serially diluted standard, and the target gene expressions were normalized by the level of GAPDH expression in each individual sample.

### DNA-binding ELISA for nuclear p65

RAW 264.7 cells (0.5 × 10^6^) cells were plated into 24-well plates. The cells were collected 10 min after stimulation with rRANKL or MHP1, and the nuclear extracts were prepared using a Nuclear Extract Kit (Active Motif, Carlsbad, CA, USA). Expression of nuclear p65 was quantified using TransAM^®^ NFκB Transcription Factor ELISA Kits (Active Motif, Carlsbad) according to the manufacture’s protocol.

### Immunohistochemical staining

The mice were perfused with 4% PFA, and the brains were cut into 12-μm thick sections. These sections were fixed and then blocked. The sections were incubated with anti-F4/80 (1:50; AbD Serotec, Oxford, UK). Then, the sections were incubated with an anti-rat fluorescent antibody (1:500 for F4/80 and CD11b, Alexa Fluor 488; Invitrogen). The immunohistochemical staining was examined using a fluorescence microscope (FSX-100; Olympus).

### Analysis of the osteoclast precursor cell differentiation

Osteoclast precursor cells were purchased from COSMO BIO (Tokyo, Japan). The cells were cultured, as described previously^27^. Briefly, the cells were seeded in 48-well plates and incubated in α-MEM (Wako, Osaka) with mouse M-CSF (50 ng/ml; R&D Systems). Mouse rRANKL (50 ng/ml; Peprotech) and/or MHP1 (30, 50, 100 μg/ml) was added simultaneously to 6 wells of each group. TRAP staining was performed using a TRAP staining kit (COSMO BIO). After 7 days of cultivation, micrographs were taken (BZ-9000; Keyence, Tokyo), and the numbers of TRAP-positive multinucleated cells (>3 nuclei) were counted as osteoclasts.

### Surgical procedure

The plan of animal studies was approved by the Animal Committee of Graduate School of Medicine, Osaka University (25-029-012), and all animal experiments were carried out in accordance with the guidelines of Osaka University. All surgeries were performed under isoflurane, and all efforts were made to minimize suffering. The C57/Bl6/J mice were obtained from CLEA Japan, Inc. The transient middle cerebral artery occlusion procedure was described previously (1). Briefly, the mice were anaesthetized with isoflurane (1.4%). The cerebral blood flow was measured using a laser Doppler flowmeter (Unique Acquisition software; Unique Medical, Osaka, Japan). A 6.0 monofilament surgical suture was advanced into the internal carotid artery to obstruct the origin of the middle cerebral artery. The filament was left in place for 40 min and then withdrawn. For all the mice, the rectal temperature was maintained at 37.0 ± 0.5 °C during surgery and recovery period, until the animals regained consciousness. Only animals that exhibited a typical reduction pattern and >82% reduction in the CBF during MCAo (in which CBF recovered by 30–80% after 5 min of reperfusion) and modified Bederson scale^28^ 1 or 2 at 4 hours after ischemia were included in the study. MHP1 (2 mg/ml in water) was diluted to 312.5 μg/ml in aCSF, and 2 μl of the MHP1 was injected intracerebroventricularly 4 hours after the MCAo. As vehicle controls for MHP1, the solvent was injected similarly. In the group of edaravone-treated mice, edaravone (3 mg/kg, i.p., NIPRO, Osaka, Japan) was injected after intracerebroventricular injection of the solvent 4 hours after the MCAo. Edaravone was repeatedly administered twice per day according to the previously reported method^29^. As vehicle controls for edaravone, saline was injected similarly. The body temperature was measured at 4, 8, 24, 48, and 72 hours after an ischemic insult by rectal probe thermometer (Unique Medical) without anaesthesia. Although 51 mice were exposed to MCAo, 17 mice (33.3%) were excluded before injection because of poor reduction in CBF or quick recovery in neurological deficit (Bederson score = 0) at 4 hours after MCAo. Two mice died at 2 days after MCAo; one in MHP1-vehicle control group (11.1%) and one in MHP1 group (11.1%). There was no death both in edaravone and edaravone-vehicle control group. The ischemic damage was evaluated at 72 hours after MCAo in sections stained with cresyl violet. Coronal sections (12 μm thickness) were made at –1.4, –0.7, 0, 0.7 and 1.4 mm from the bregma, mounted on the stereomicroscope and photographed. The corrected hemispheric lesion area (HLA) was calculated as HLA (%) = [LT − (RT − RI)]/LT × 100, where LT is the area of the left hemisphere, RT is the area of the right hemisphere, and RI is the infarcted area.

Core body temperature in normal mice was continuously recorded using temperature data loggers (KN Laboratories, Osaka, Japan) implanted into the peritoneal cavity, as described before^6^. The data loggers were programmed to record body temperature every 2 min with a resolution of 0.1 °C. Following the implant, animals were allowed 1 day to recover.

### Statistical analysis

All values are expressed as the means ± SD. Multiple comparisons were evaluated by ANOVA followed by Tukey’s multiple comparisons test. The differences in body temperature were analysed two-way ANOVA followed by Tukey’s multiple comparisons test. Two groups were compared using the unpaired t-test (Fig. 8A–D). Differences were considered to be significant at *P* < 0.05.

## Additional Information

**How to cite this article**: Kurinami, H. *et al*. A Novel Therapeutic Peptide as a Partial Agonist of RANKL in Ischemic Stroke. *Sci. Rep.* **6**, 38062; doi: 10.1038/srep38062 (2016).

**Publisher's note:** Springer Nature remains neutral with regard to jurisdictional claims in published maps and institutional affiliations.

## Supplementary Material

Supplementary Information
