# Supplementary material for: A Novel Therapeutic Peptide as a Partial Agonist of RANKL in Ischemic Stroke
Source: Sci Rep. 2016 Nov 29;6:38062. doi: 10.1038/srep38062 (PMC5126682; doi:10.1038/srep38062)
Supplement: Supplementary Information [file srep38062-s1.pdf]

A Novel Therapeutic Peptide as a Partial Agonist of RANKL in Ischemic Stroke

Hitomi Kurinami[1,2], Munehisa Shimamura[1,3*], Hironori Nakagami[1], Hideo Shimizu[4], Hiroshi Koriyama[1], Tomohiro Kawano[3], Kouji Wakayama[5], Hideki Mochizuki[3], Hiromi Rakugi[6], Ryuichi Morishita[7*]

1. Department of Health Development and Medicine, Osaka University Graduate School of Medicine, Japan
2. Postgraduate Medical Training Centre, Osaka University Hospital, Japan
3. Department of Neurology, Osaka University Graduate School of Medicine, Japan
4. Department of Nutritional Science, Kansai University of Welfare Sciences, Japan
5. Department of Advanced Clinical Science and Therapeutics, Graduate School of Medicine, the University of Tokyo, Japan
6. Department of Geriatric and General Medicine, Osaka University Graduate School of Medicine, Japan
7. Department of Clinical Gene Therapy, Osaka University Graduate School of Medicine, Japan

Corresponding authors: Ryuichi Morishita[1], Munehisa Shimamura[2,3]

[1]Department of Clinical Gene Therapy, [2]Department of Health Development and Medicine and [3]Department of Neurology, Osaka University Graduate School of Medicine, Centre of Medical Innovation and Translational Research (6th floor, Room 0612B), Osaka University, 2-2 Yamada-oka, Suita, Osaka 565-0871, Japan

Tel: +81-6-6210-8359; Fax: +81-6-6210-8360

Email: morishit@cgt.med.osaka-u.ac.jp; shimamuu@cgt.med.osaka-u.ac.jp

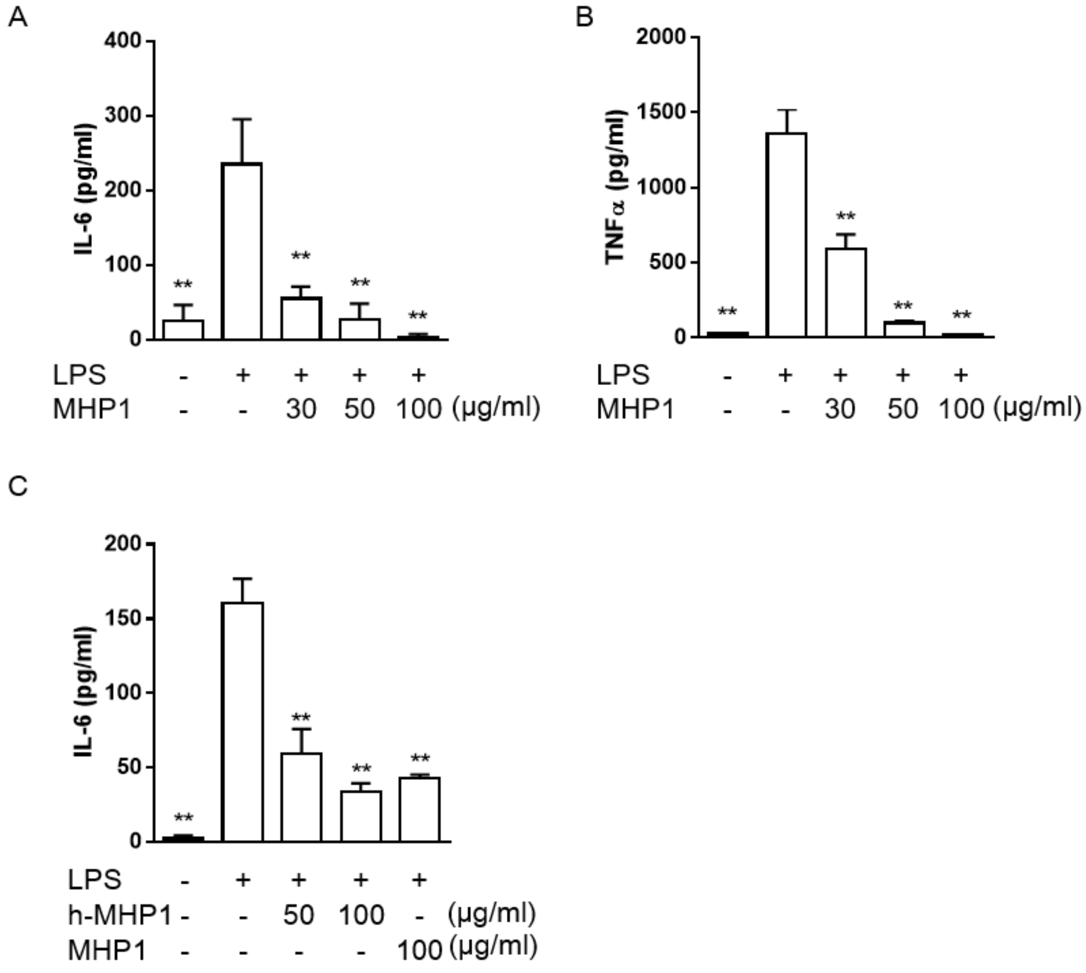

Supplementary Figure S1. The effects of MHP1 and h-MHP1 in RAW 264.7 cells or THP1 cells.

MHP1 effectively inhibited the LPS-induced cytokine expression in RAW 264.7 cells (A, B). h-MHP1 suppressed LPS-induced IL-6 expression in THP1 cells, but MHP1 also inhibited cytokine expression (C). ***P*<0.01 vs. the LPS-treated group. N = 3 in each group.

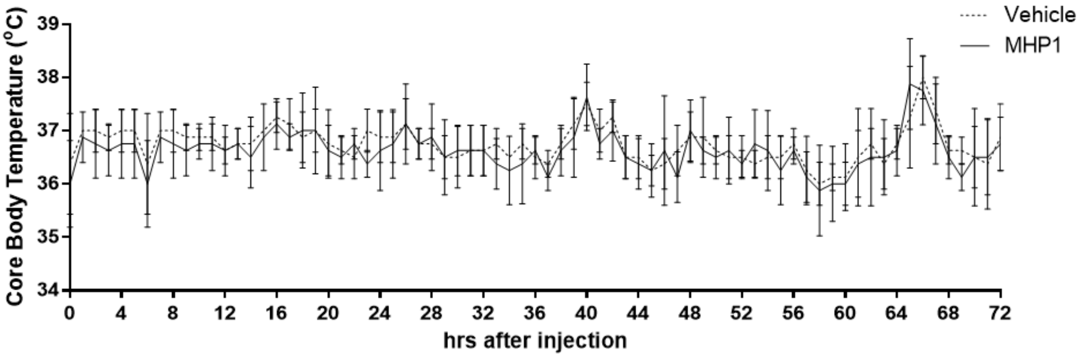

Supplementary Figure S2. The effects of MHP1 on core body temperature in normal mice

MHP1 was injected intracerebroventricularly in normal mice. The core body temperature was continuously monitored for 3 days. N = 4 in each group.

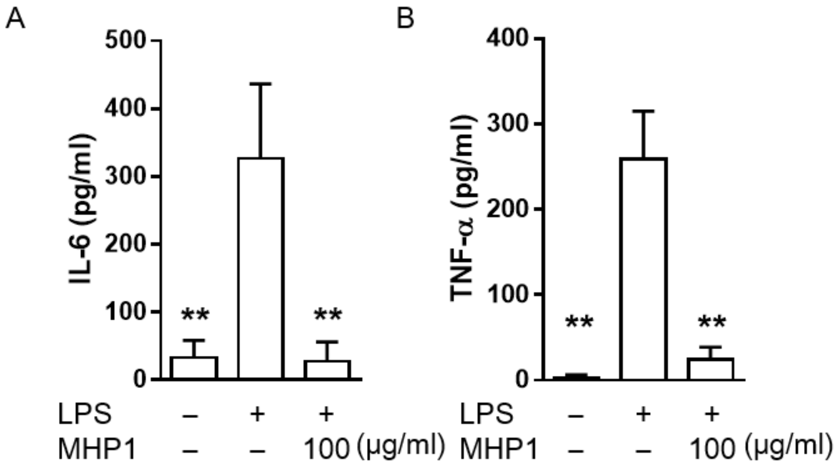

Supplementary Figure S3. The effects of MHP1 stored at 4°C for 6 months on LPS-stimulated MG6 cells.

MHP1 was dissolved in ddH_2O at a concentration of 1 mg/ml and stored at 4 °C for 6 months. The stored solution was added to the culture medium with LPS. Stored MHP1 significantly reduced cytokine expressions. ***P*<0.01 vs. cells treated with LPS alone. N = 4 in each group.
